# Neoadjuvant chemotherapy for stage II–III breast cancer: a single-center experience

**DOI:** 10.1186/s12957-023-03199-z

**Published:** 2023-10-07

**Authors:** Haidi Abd El Zaher, Hamada Fathy, Mohamed Abozeid, Mohammed Faisal

**Affiliations:** 1 Surgical Oncology Unit, Department of Surgery, Faculty of Medicine, Suez Canal University Hospital, Ismailia, Egypt; 2 Department of Oncology and Nuclear Medicine, Faculty of Medicine, Suez Canal University, Ismailia, Egypt; 3 Department of General Surgery, Torsby Hospital, Region Värmland County Council, Värmland, Sweden

**Keywords:** Neoadjuvant chemotherapy, Breast-conserving surgery, Breast cancer, Mastectomy

## Abstract

**Introduction:**

We conducted this study to reflect a single-center experience with the use of neoadjuvant systemic chemotherapy (NAC) for the management of women with operable breast cancer.

**Methods:**

We conducted a retrospective chart review on all women presenting with operable, stage II–III, breast cancer and were scheduled for NAC at Suez Canal University Hospital. The primary outcome of this study was to estimate the proportion of patients with breast cancer who become eligible for breast-conserving surgery (BCS) after (NAC).

**Results:**

A total of 147 patients were included. Before the initiation of chemotherapy, only 66 (44.9%) patients were indicated for (BCS). A total of 40 (49.4%) new patients, out of the 81 patients who were ineligible before chemotherapy, became eligible for BCS after NAC (95% CI 39.3–61.9%). On the other hand, 8 (12.1%) patients became ineligible for BCS after NAC, out of 66 patients who were initially eligible. Out of the 98 eligible patients for BCS after chemotherapy, 72 (73.5%) patients underwent the surgery, and the remaining 26 (26.5%) patients chose modified radical mastectomy (MRM). A total of 55 out of 72 (76.4%) patients achieved pathological complete response (pCR). One woman (0.1%) experienced relapse in the 3rd year of follow-up and three women (2%) experienced relapse in the 5th year of follow-up. We found a statistically significant relationship between patients who became eligible for breast-conserving surgery and both age and estrogen receptor negativity (*p* = 0.001 and 0.007, respectively).

**Conclusion:**

NAC can play a crucial role in increasing the rate of eligibility for BCS among women with operable, stage II–III, breast cancer.

**Supplementary Information:**

The online version contains supplementary material available at 10.1186/s12957-023-03199-z.

## Introduction

There are several different types of patients who present with locally advanced breast cancer (LABC), and their survival and rates of local recurrence can vary. The description of this form of breast cancer is not universally accepted, although one widely used clinical staging comprises individuals with big original tumors larger than 5 cm (T3), fixed cutaneous or chest involvement (T4), fixed axillary (N2), or ipsilateral internal mammary lymph node involvement [1]. According to the 8th TNM staging system proposed by the American Joint Committee on Cancer (AJCC), all stage III disease is therefore considered locally advanced, as is a subset of stage IIB (T3N0) [2]. In the 1970s, the original treatment approaches of surgery, radiation, or all of them were revised to entail a multimodality approach that includes neoadjuvant chemotherapy because they had little effect on the survival rates of these patients [3].

Even if the outcomes of utilizing neoadjuvant chemotherapy in operable breast tumors point to a potential rise in breast-conserving rates, survival is identical to those of postoperative adjuvant chemotherapy. The primary objective of neoadjuvant treatment is to achieve resectability, either through a normal mastectomy or breast-conserving surgery, as patients with LABC frequently have an inoperable illness at diagnosis [4].

Neoadjuvant chemotherapy had many advantages over the conventional approach of surgery followed by adjuvant chemotherapy. Considerable evidence has stated that neoadjuvant systemic chemotherapy has improved overall survival (OS) rates and disease-free survival (DFS) [3, 5–7]. It significantly decreases lymph nodal metastasis and primary tumor size in most cases; thus, it can increase the utility of breast-conserving surgery, which improves body appearance and sexual function in comparison to mastectomy [8–12]. A recent meta-analysis, involving 10 studies with 4756 patients, reported that neoadjuvant chemotherapy has increased breast-conserving surgery from 49 to 65% compared to adjuvant therapy [13]. A recent prospective study, enrolling 634 patients, reported that neoadjuvant systemic therapy facilitated BCS in 53.2% of women with triple-negative breast cancer [14]. Another report noted that 27% of patients who were appropriate for mastectomy underwent breast-conserving surgery and their breast cancer recurrence rates were 14.3% [8]. Besides, NAC has provided good results regarding tumor recurrence rates in women with breast cancer; the breast cancer recurrence rates of patients appropriate for breast-conserving surgery at the outset were 6.9% [15]. Both groups had comparable rates of overall survival and disease-free survival [15].

Breast cancer subtypes are categorized by molecular markers such as the human epidermal growth factor receptor 2 (HER2), progesterone receptor (PR), and estrogen receptor (ER), and each subtype has a unique behavior and response to chemotherapy [16, 17].

After NAC, the (pCR) in the breast and axillary lymph node would improve outcomes, and it is utilized by some populations as a substitute survival metric [18, 19].

Based on tumor biologic subgroups, some studies have demonstrated pCR rates with some fluctuation up to 40% after NAC [7, 10–12]. Triple-negative (TN) tumors had the highest pCR rate and excellent outcomes, followed by HER 2-positive tumors, while hormone-positive tumors have the lowest rates pCR [20].

There are a number of constraints that contribute to the reported variances in the pCR, including the non-standard definition of the pCR, the existence of non-invasive and invasive cancer, the prognostic significance of breast cancer subtypes, and variations in NAC regimens.

We conducted this study to assess the importance of NAC in increasing the possibility of undergoing BCS among different molecular subgroups of stage II–III breast cancer at Suez Canal University Hospital.

## Materials and methods

The protocol of the current study was approved by the local ethics committee of Suez Canal University Hospital. The need for signed informed consent was waived due to the retrospective nature of the study. All procedures run in compliance with the standards of the Declaration of Helsinki [21]. We prepared the following manuscript in concordance with STROBE guidelines [22].

### Study design and patients

We conducted a retrospective chart review on all women presenting with operable, stage II–III, breast cancer and were scheduled for NACat Suez Canal University Hospital through the period from January 2019 to February 2021. Only women whose breast cancer diagnosis was confirmed with a biopsy and who were treatment-naïve were included. Records of patients with stage IV, inflammatory illness, those who did not complete a full course of systemic neoadjuvant therapy due to disease progression or medication toxicity, male breast cancer, and other neoadjuvant treatment modalities like radiotherapy were also excluded.

### Study’s procedures

From the patient’s medical records, the following information was collected: age, sex, tumor multifocality, size and stage menopausal status, pathological types included invasive ductal carcinoma (IDC) and special type of breast cancer such as invasive lobular carcinoma, and invasive NOS, histological grade, and NAC regimen, local invasion, the eligibility for surgery, and the pre-chemotherapy type of surgical decision and pathological outcomes. Every patient would have mammogram (MMG), a breast ultrasound sonography (USG) and MRI after NAC assessment. A contrast CT of the chest and abdomen was used for the metastatic workup. When a physical examination and ultrasonography revealed that a lymph node was positive (classified as N0 and N1–2), the lymph node status was determined by core needle biopsy (CNB). Last but not least, breast pathologists carried out the diagnostic biopsy and assessed the removed tissues. Immunohistochemical (IHC) staining was used to assess the expression of ER, PR, HER2, and Ki67; a level of ER and PR less than 1% was regarded as negative; the lack of both ER and PR expression was defined as HR-negative; and the presence of either was classified as HR-positive [23].

IHC analysis or amplification confirmed by FISH categorized HER2 positivity as 3+; values 0 or 1+ were considered HER2-negative [24]. After assessing the complete section, 1000 invading cancer cells with a representative area, not less than 500 cancer cells, were chosen for counting Ki67. The Ki67 expression was then split into two groups, Ki67 > 10% and Ki67 10%, in accordance with the ideal cutoff values. The molecular subtypes included four categories: luminal A (er+ and pr +/her2), luminal B subtype (er+ and/or pr+, any her2−, Ki67 > 10%), her-2 enriched subtype (er−, pr−, her2+), and triple-negative subtype (er−, pr−, her2−) [25].

At our center, a surgical specialist determines the eligibility for breast conservative surgery according to the tumor size, multi-centricity, presence of diffuse calcification, the presence of nipple invasion or tumors located close to the nipple, and the expected cosmetic result. All women who were deemed eligible for surgery received neoadjuvant systemic treatment. The regimen was 4 cycles of doxorubicin/cyclophosphamide, followed by 4 cycles of paclitaxel for all participants. Trastuzumab was added for patients with human epidermal growth factor receptor 2 (HER2)-positive breast cancer. For patients with triple-negative breast cancer (TNBC), we added carboplatin to paclitaxel. Six weeks later, breast cancer surgery was performed. The choice of BCS or MRM was based on surgeon/patient’s decision. Regarding axilla care, axillary dissection will be carried out regardless of the axillary response to NAC if the axillary lymph node was determined to be metastatic on cytology/histology prior to surgery; otherwise, sentinel lymph node biopsy (SLNB) will be carried out during breast surgery.

We assessed the overall pathologic response in both the breast and axilla. The most common definition of overall (pCR) was no evidence of residual invasive cancer in either the breast or axilla. No matter how much the breast or axilla changed, a partial reaction (PR) was taken into account if there was any response. If there were no changes and signs of regression in the breast and axilla, no reaction (NR) was recorded, and we relied on these definitions.

### Study’s outcomes

The primary outcome of this study was to estimate the proportion of patients with operable breast cancer who become eligible for BCS after NAC. The choice of post-chemotherapy surgery was based on the surgeon/patient’s decision or when the BCS was deemed unsuccessful intraoperatively. The secondary outcomes of this study were the rate of pCR after BCS and the rate of recurrence after surgery.

### Statistical analysis

We employed descriptive statistics to describe the patients’ age, menopausal status, stage, grade, immunohistochemical results, the pre-chemotherapy type of surgery, contradictions for BCS, type of chemotherapy, the post-chemotherapy contradictions for BCS, the success rate of breast conservative surgery, and recurrence after BCS. The proportion of the patients with operable breast cancer who become eligible for BCS after NAC was calculated with 95% confidence intervals (CI) using exact binomial methods. Retrieved data were processed with IBM SPSS statistical software (version 25).

## Results

A total of 147 patients were included, with a mean age of 47.7 ± 10.5 years old (minimum/maximum was 25/60 years old). Of them, 45 (30.6%) were post-menopause. The most commonly encountered pathological grade was grade III (49.7%), followed by grade II (38.8%). Invasive ductal carcinoma accounted for the vast majority of the cases (89.8%). Based on the 8th TNM staging system recommended by the AJCC, the IIB (43.5%) and IIIA (25.9%) stages were the most common stages. Nearly 14.28% of the patients were luminal A, 26.53% were luminal B, and 36.7% of the patients were HER-2 positive and nearly 22.44% were triple negative (Table 1).

**Table 1:** Distribution of the studied patients by their demographic and clinicopathological

Variables	Patients (*n* = 147)
Age in years, mean ±SD	47.7 ± 10.5
< 40 years	98 (66.7%)
> 40 years	49 (33.3%)
Menopausal status, no. (%)	
- Premenopausal	102 (69.4%)
- Postmenopausal	45 (30.6%)
Pathological grade, no. (%)	
- Grade I	14 (9.5%)
- Grade II	57 (38.8%)
- Grade III	73 (49.7%)
- Unknown grade	3 (2%)
Histological type, no. (%)	
- Ductal	132 (89.8%)
- Lobular	4 (2.7%)
- Invasive, nos.	9 (6.1%)
- Other	2 (1.4%)
TNM stage, no. (%)	
IIA	28 (19.0%)
IIB	64 (43.5%)
IIIA	38 (25.9%)
IIIB	17 (11.6%)
Hormonal receptors, no. (%)	
- ER-positive	94 (63.9%)
- PR-positive	62 (42.2%)
- Her-2-positive	54 (36.7%)
BC subtypes, no. (%)	
Luminal A	21 (14.3%)
Luminal B	39 (26.5%)
HER 2 +ve	53 (36.1%)
Triple negative	34 (23.1%)
KI67 index, no. (%)	
< 10	45 (41.5%)
> 10	86 (58.5%)

**Fig. 1:**
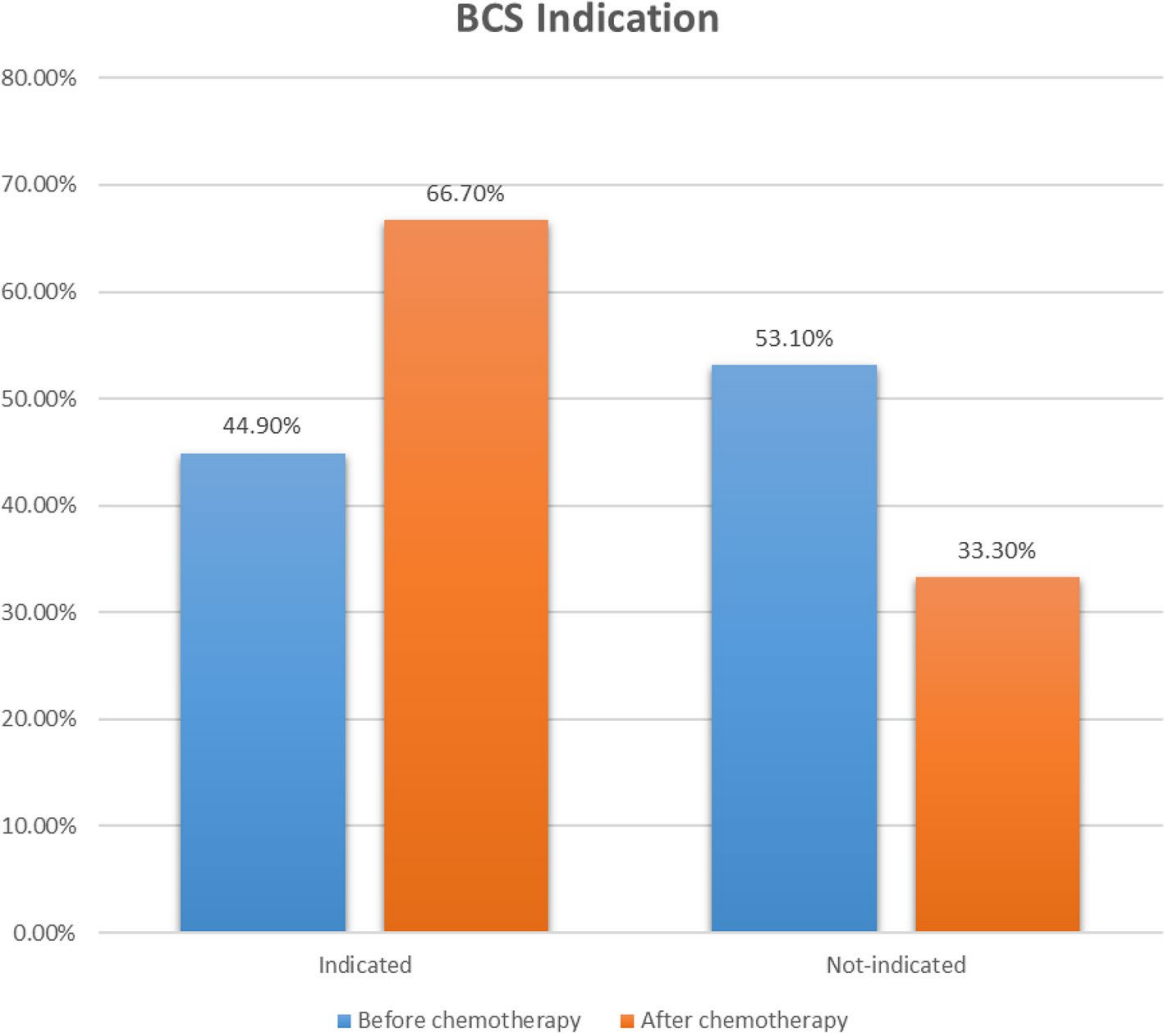
Distribution of studied patients by their eligibility for BSC before and after chemotherapy

Before the initiation of chemotherapy, only 66 (44.9%) patients out of 147 patients were indicated for breast-conserving surgery (Fig. 1). The most common contraindications for breast-conserving surgery were large tumor size (48.1%), multi-centricity (18.5%), and poor cosmetic outcome (14.8%). The distribution of chemotherapeutic regimens was as the following: Doxorubicin and Cyclophosphamide (AC) followed by paclitaxel (38.1%) in luminal breast cancer, AC plus paclitaxel plus trastuzumab (36.7%) in her2 positive, and AC plus paclitaxel plus carboplatin (25.2%) triple-negative. After the completion of the chemotherapy regimen, 98 (66.7%) of the women were deemed eligible for breast-conserving surgery (Fig. 1). A total of 40 (49.4%) new patients, out of the 81 patients who were ineligible before chemotherapy, became eligible for breast-conserving surgery after neoadjuvant chemotherapy (95% CI 39.3–61.9%). On the other hand, 8 (12.1%) patients, out of the 66 patients who were eligible before chemotherapy, became ineligible for breast conservative surgery after neoadjuvant chemotherapy. Thus, 98 patients were eligible for breast-conserving surgery after chemotherapy. Of them, 72 (73.5%) patients underwent the surgery, and the remaining 26 patients chose modified radical mastectomy (Tables 2 and 3). On the other hand, the causes of refusing breast-conserving surgery in the 49 ineligible patients were large tumor size (14.3%) or high recurrence risk (diffuse micro-calcifications or multi-centricity (81.6%), or patients’ choice (4.1%).

**Table 2:** Distribution of studied patients according to their BCS eligibility and final surgical decisions

	Total (*n* = 147)	Remained BCS	Converted to BCS	Converted to mastectomy
BCS candidate pre-chemotherapy	66 (44.9%)	58 (87.9%)	–	8 (12.1%)
BCS ineligible before chemotherapy	81 (55.1%)	–	40 (49.4%)	41 (50.6%)
Final surgery decision				
- BCS	98 (66.7%)	72 (73.5%)	–	26 (26.5%)
- MRM	49 (33.3%)	–	–	–

**Table 3:** Distribution of studied patients according to changes in eligiblity for BCS before and after chemotherapy

		After chemotherapy		Total
		Eligible	Ineligible	
Before chemotherapy	Eligible (Row%, Grand Total%)	58 (87.9%, 39.5%)	8 (12.1%, 5.4%)	66 (100%, 44.9%)
	Ineligible (Row%, Grand Total%)	40 (49.4, 27.2%)	41 (50.6%, 27.9%)	81 (100%, 55.1%)
Total		98 (66.7%)	49 (33.3%)	147

**Fig. 2:**
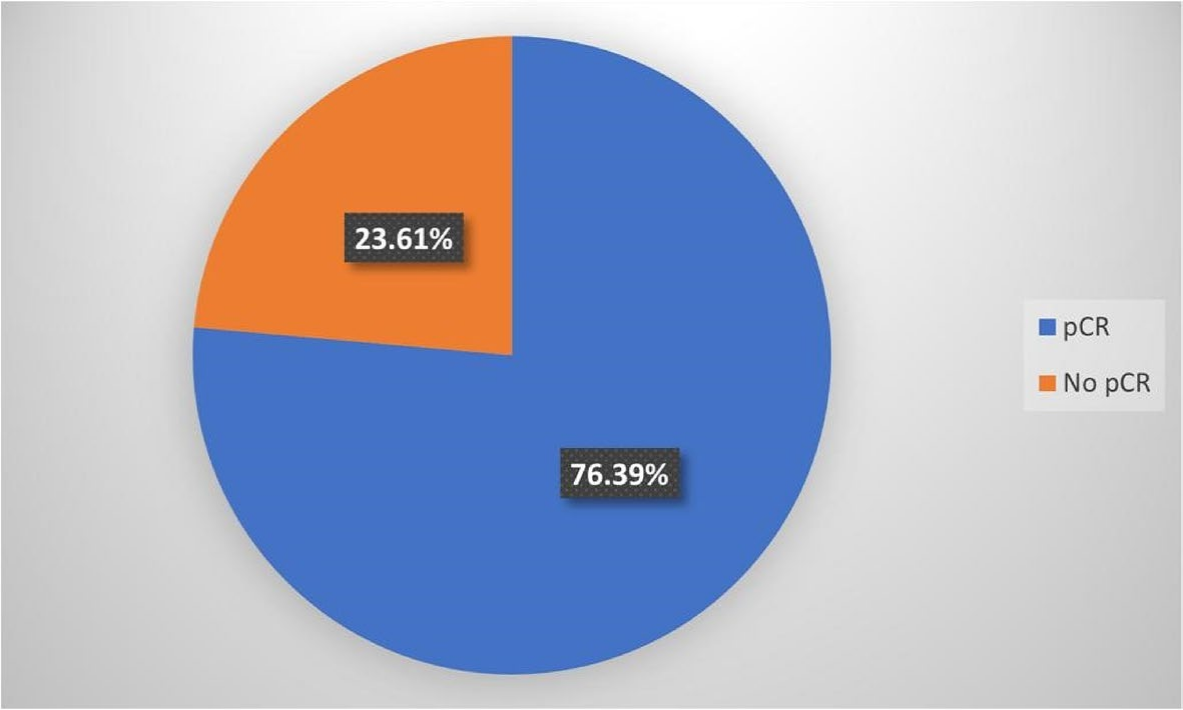
Distribution of patients who underwen conservative surgery according to pCR

**Fig. 3:**
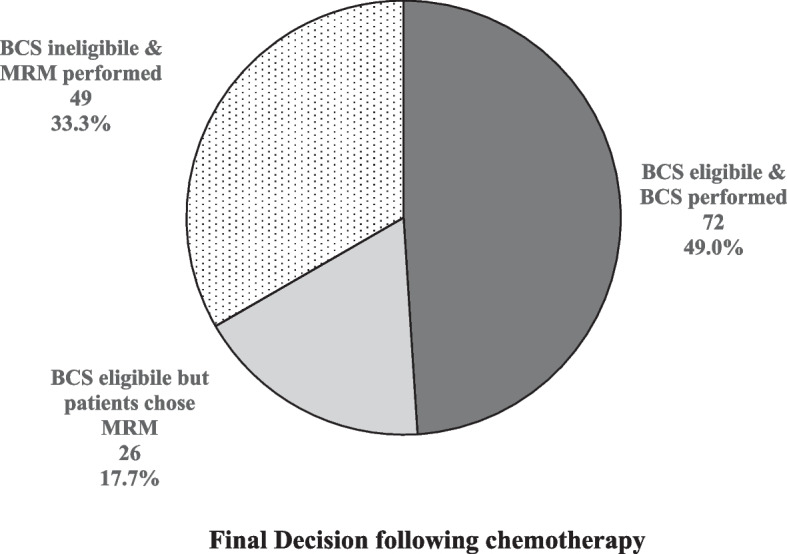
Distribution of the studied patients according to the final surgical decisions following chemotherapy

Out of the 72 patients who underwent conservative surgery, a total of 55 (76.4%) patients were found to have pCR (Figs. 2 and 3). Axillary treatment for patients who received BCS included 15 patients (10.2%) SLNB and 57 patients (38.7%) ALND (Table 4). Out of the 72 patients who underwent conserving surgery, a total of 55 (76.4%) patients were found to have pCR. On the other hand, only 31 cases (41.3%) of the mastectomy group achieved pCR (Table 5). pCR existed in higher percentages in cases with Ki67 above 10% (52.7%) and when trastuzumab or carboplatin was added to the AC, however, no statistically significant associations were detected.

**Table 4:** Axillary treatment in the BCS group

Axillary treatment	SLND	ALND
*N* (%)	15 (10.2%)	57 (38.7)

**Table 5:** The prevalence of patients with pCR in BCS and mastectomy groups

pCR	BCS	Mastectomy
Overall pCR in both breast and axilla	55 (76.4%)	31 (41.3%)

On the other hand, pCR was significantly associated with the BC molecular subtypes, where HER-2-positive and triple-negative subtypes showed the highest percentages of pCR (77.4% and 61.8%, respectively) (Table 6).

**Table 6:** Distribution of the status of complete pathological response by the BC subtypes among the studied breast cancer patients

		Yes		No		*p* value
		Count	Row *N* %	Count	Row *N* %	
BC subtypes	Luminal A	6	28.6%	15	71.4%	< 0.001*
	Luminal B	18	46.2%	21	53.8%	
	HER 2 +ve	41	77.4%	12	22.6%	
	Triple-negative	21	61.8%	13	38.2%	
	Total	86	58.5%	61	41.5%	

Chi-square test

*statistically *p*-value at below 0.05

Overall, one woman (0.1%) experienced local recurrence at the 3rd year of follow-up and three women (2%) experienced recurrence at the 5th year of follow-up despite receiving adjuvant radiotherapy after having BCS. Of these four patients, three were ineligible for breast-conserving surgery before chemotherapy. No recurrence was observed in the mastectomy group. The difference between patients who underwent breast-conserving surgery and MRM was not statistically significant (*p* = 0.22 and 0.07, respectively). Unfortunately, since no patient experienced recurrence in the total mastectomy group, thus, presenting disease-free survival (PFS), comparing the two groups was not feasible from a statistical point of view. Thus, we calculate the PFS for the BCS group only.

Patients, who were deemed eligible for BCS after chemotherapy, were significantly older than ineligible patients (*p* = 0.019). Ineligible patients were more likely to have a higher grade (*p* = 0.007), higher TNM stage (*p* < 0.001), HER-2-negative (*p* = 0.004), higher Ki67 index (*p* = 0.001), larger tumor size (*p* = 0.001), more diffuse micro-calcification (*p* = 0.001), and were more likely to receive AC plus paclitaxel only (*p* = 0.008).

We performed a multivariate regression analysis for predicting eligibility of patients or BCS after NAC. Explanatory variables included the patient’s age, menopausal state, hormonal receptor expression, grade and stage of tumor, type of tumor, and type of chemotherapy. A significant relation between the patients who become eligible for BCS, and age was found (Table 6). The younger the patients, the more they become eligible for BCS (*p* = 0.001). This was also found with the menopausal status; premenopausal women were more eligible than postmenopausal women by 1.256 (*p* = 0.034). The ER-negative patients were more eligible for BCS than ER-positive patients by 3.315 (*p* = 0.007) (Table 7).

**Table 7:** Multivariate regression analysis for predicting eligibility of patients for BCS after neoadjuvant chemotherapy

		*B*	Std. error	Wald	Sig.	Exp(B)	95% confidence interval for Exp(B)	
							Lower bound	Upper bound
BCS	Age	− 0.119	0.036	10.751	0.001*	0.887	0.826	0.953
	Menopause (no)	− 1.256	0.591	4.511	0.034*	0.285	0.089	0.908
	Grade (1vs4)	0.932	0.520	3.215	0.073	2.539	0.917	7.029
	Type (1vs6)	− 0.455	0.507	.803	0.370	0.635	0.235	1.715
	Stage (1vs7)	− 0.362	0.264	1.874	0.171	0.696	0.415	1.169
	ER (no)	− 3.315	1.224	7.339	0.007*	0.036	0.003	0.400
	PR (no)	1.390	0.772	3.241	0.072	4.016	0.884	18.248
	HER2 (no)	0.365	0.748	.237	0.626	1.440	0.332	6.243
	KI67	0.469	0.456	1.056	0.304	1.598	0.654	3.910
	Chemotherapy	− 1.676	0.656	6.538	0.011	0.187	0.052	0.676

The full model is significant at 0.000

*statistically *p*-value at below 0.05

## Discussion

The results of this indicated that the neoadjuvant chemotherapy led to a notable increase in the proportion of breast cancer patients who performed breast-conserving surgery. Besides, more than two-thirds of the patients who underwent breast-conserving surgery achieved pCR. The conversion of the patients to breast-conserving surgery after neoadjuvant chemotherapy did not have a negative impact on the recurrence rate as well. The comparative analysis showed that patients who responded to neoadjuvant chemotherapy were more likely to be younger, HER-2-positive, and had lower tumor grade and stage.

Breast-conserving surgery (BCS) is the modality of choice for early breast cancer (stage I–II) due to its comparable survival benefits and better cosmetic outcome, compared to radical surgery [26]. In patients with more advanced carcinoma, a relatively lower proportion of the patients become eligible for BCS, who are usually young patients with small localized tumor and favorable physical status [27]. Neoadjuvant chemotherapy has the advantages of down-staging tumor size before surgery permitting less-invasive surgery. An additional advantage of neoadjuvant chemotherapy is its ability to aid intraoperative tumor recognition and reduce the possibility of extensive residual disease [28, 29]. Several studies have assessed the efficacy in increasing the rate of breast cancer patients eligible for conserving surgery. In this report, the use of neoadjuvant chemotherapy increased the proportion of women eligible for breast-conserving surgery by 50%. In line with these findings, Debled et al. [30], noted that 71% of HER2-positive patients underwent breast-conserving surgery after neoadjuvant chemotherapy. The same results were obtained by Semiglazov et al. [30], Vergine et al. [31], and Cho et al. [32]. In Golshan et al. [33], 42% of patients who were initially deemed ineligible were converted by neoadjuvant chemotherapy to BCS. In a previous meta-analysis, the neoadjuvant chemotherapy led to breast-conserving surgery in nearly 65% of the patients [13]. However, there is another study that shows controversy regarding the positive impact of neoadjuvant chemotherapy on the rate of breast-conserving surgery. According to Boughey et al. [34], the neoadjuvant chemotherapy did not increase the rate of BCS among women with invasive breast cancer.

Higher pCR can intuitively favor the decision of performing breast-conserving surgery, in patients who were previously candidates for mastectomy [35]. Previously, it was found that patients with pCR had better survival outcomes than patients without pCR and underwent BCS [32]. In this report, we found that out of the 72 patients who underwent conserving surgery, a total of 55 (76.4%) patients were found to have pCR. Higher pCR was observed with ER−, HER+, Ki67 > 10% and when trastuzumab or carboplatin was added to the AC. According to research by Boughey et al., individuals with triple-negative disease (38.2 %) and HER2-positive disease (45.4 %) had overall pCR that was considerably greater than patients with HR+/HER2− (11.4%) [36]. In the German population, the von Minckwitz et al. study revealed pCR rates of 8.9% for luminal A and 15.4% for luminal B/HER2− illness (*n *= 1994 for these two categories) [37]. When the analysis is limited to HR+/HER2− tumors, Lips et al.’s research has demonstrated that lobular histology was not linked with chemotherapeutic response [38].

Local recurrence is a major concern in patients undergoing breast-conserving surgery, previous reports indicated that up to 15% of the patients undergoing BCS will develop locoregional recurrence [39]. However, the application of neoadjuvant chemotherapy can potentially reduce the risk of recurrence. In a recent meta-analysis, the rate of recurrence in patients who received neoadjuvant chemotherapy and breast-conserving surgery was 9.2%, compared to 8.3% in the mastectomy group [40]. In the present study, we found that the 5-year local recurrence-free rate was 98%; the difference between patients who underwent breast-conserving surgery and modified radical mastectomy was not statistically significant. Our findings run in line with the results of NSABP-B18 trial [9] and other studies [5, 41]. The study by Ishitobi et al. [42], also found no difference in local recurrence-free rate according to type of surgery after neoadjuvant chemotherapy in patients with a planned mastectomy at the initial exam.

When considering the scope of this data on breast tumor response and patient outcome various clinical implications become feasible. To begin, breast tumor response may serve as a valuable signal for preoperative therapy on micro-metastases and survival outcome because of its correlation with subsequent prognosis. The second benefit is that because preoperative therapy has the same overall effect as postoperative therapy, more research using preoperative therapy can be conducted without worry of harming patients. It is possible to assess the response of a breast tumor to preoperative therapy within a matter of weeks, allowing for early judgments to be reached about the relative utility of novel chemotherapeutic regimens alone, in combination, or in sequence with those that have already demonstrated efficacy.

We acknowledge the existence of certain limitations of the present study. The current study was a retrospective chart review, with the inherited limitations of misclassifications, missing data, or recording errors. Besides, the results of genetic testing were not available for all patients, which might have affected the choice of surgery. There were no clear data regarding the causes underlying patients’ choice to undergo total mastectomy, despite being eligible for conservative surgery. The choice of surgery was based mainly on the surgeon’s decision, which may introduce biases in the decision-making process. Lastly, all patients underwent breast magnetic resonance imaging (MRI) after neoadjuvant chemotherapy, which could have increased the rate of mastectomy in the present study; previous studies reported that MRI was a major factor for the increased rate of mastectomy due to its ability to detect additional abnormalities [43].

In conclusion, neoadjuvant chemotherapy can play a crucial role in increasing the rate of eligibility for breast-conserving surgery among women with operable, stage II–III, breast cancer. Our findings indicated that employing neoadjuvant chemotherapy can increase the rate of eligible breast-conserving surgery by nearly 50%. The pCR after breast-conserving surgery in our study was notably high. Besides, we also found that the use of breast conservative surgery after the neoadjuvant chemotherapy did not significantly increase the risk of recurrence. Given the scarcity in the published literature, it is recommended to conduct rigorous trials, with a large sample size and multinational collaboration, to reflect the clinical usefulness of neoadjuvant chemotherapy in the setting of operable breast cancer.

### Supplementary Information

**Additional file 1.**

## Data Availability

The corresponding author can provide the datasets used and/or analyzed during the current investigation upon reasonable request.

## Abbreviations

AC: Adjuvant chemotherapy
ALND: Axillary lymph node dissection
BCS: Breast-conservative surgery
BC: Breast cancer
CI: Confidence intervals
CT: Computed tomography
DFS: Disease-free survival
ER: Estrogen receptor
EFS: Event-free survival
FISH: Fluorescence in situ hybridization
HER-2: Human epidermal growth factor receptor 2
IHC: Immunohistochemical
MMG: Mammogram
MRI: Magnetic response imaging
MRM: Modified radical mastectomy
NAC: Neoadjuvant chemotherapy
NR: No reaction
OS: Overall survival
pCR: Pathological complete response
PR: Progesterone receptor
SLND: Sentinel lymph node dissection
STROBE: Strengthening the Reporting of Observational Studies in Epidemiology
TN: Triple-negative
TNBC: Triple-negative breast cancer declarations
USG: Ultrasonography
