# Supplementary material for: Neoadjuvant chemotherapy for stage II–III breast cancer: a single-center experience
Source: World J Surg Oncol. 2023 Oct 7;21:314. doi: 10.1186/s12957-023-03199-z (PMC10559473; doi:10.1186/s12957-023-03199-z)
Supplement: Supplementary file 1 — Additional file 1. [file 12957_2023_3199_MOESM1_ESM.doc]

Total Screened number

(173)

Excluded (23 Pats)

Patients with inflammatory disease.

Not receive a full course of neoadjuvant systemic treatment

Included

150 Pats

Legible for CBS before

Neo-adjuvant

66 Pats

Refused surgery

Missed during follow up.

3 Pats

Ineligible for CBS before

Neoadjuvant

81 Pats

Ineligible for CBS

8 Pats

Legible for CBS

58 Pats

Legible for CBS

40 Pats

Remained ineligible

41 Pats

41Pats

Neo-Adjuvant

Underwent CBS

72 Pats

Underwent MRM

26 Pats
